# PatternExtract: A Facile, Scalable Pipeline for Point Pattern Generation from Spatial Imaging Data

**DOI:** 10.34133/csbj.0141

**Published:** 2026-09-09

**Authors:** Shruti Sridhar, Gayatri Kumar, Victoire Ringler, Kanav Gupta, Siddham Jasoria, Ziwei Meng, Patrick Jaynes, Chartsiam Tipgomut, Vaibhav Rajan, David Scott, Claudio Tripodo, Kasthuri Kannan, Anand Devaprasath Jeyesekharan

**Affiliations:** ^1^ Cancer Science Institute of Singapore, National University of Singapore, Singapore, Singapore.; ^2^ Department of Translational Molecular Pathology, The University of Texas MD Anderson Cancer Center, Houston, TX, USA.; ^3^ Faculty of Life Science and Engineering, École Polytechnique Fédérale de Lausanne, Lausanne, Switzerland.; ^4^ Manipal Institute of Technology, Manipal, India.; ^5^ Birla Institute of Technology & Science, Pilani, India.; ^6^ British Columbia Cancer, Vancouver, BC V5Z 4E6, Canada.; ^7^ Tumor Immunology Unit, Department of Health Sciences, University of Palermo, Palermo, Italy.; ^8^ IFOM ETS, The AIRC Institute of Molecular Oncology, Milan, Italy.; ^9^ NUS Centre for Cancer Research, Yong Loo Lin School of Medicine, National University of Singapore, Singapore, Singapore.; ^10^ Department of Haematology-Oncology, National University Cancer Institute, Singapore, National University Health System, Singapore, Singapore.; ^11^ Department of Medicine, Yong Loo Lin School of Medicine, National University of Singapore, Singapore, Singapore.

## Abstract

Spatial point pattern analysis offers a mathematically rigorous framework for characterizing cell distribution in cancer tissue, yet existing pipelines lack robust methods for defining accurate spatial windows that exclude noncellular artifacts. We present PatternExtract, an open-source, multi-platform pipeline for generating biologically accurate spatial point patterns from multiplexed imaging data. The pipeline introduces a 2-kernel tissue segmentation approach that overlays concentric circular kernels on cellular coordinates to construct tissue masks without dependence on proprietary software or fluorescence composite channels, making it compatible with RGB images and coordinate outputs from any cell segmentation tool. Benchmarked on 568 images from 274 diffuse large B cell lymphoma (DLBCL) patients, PatternExtract identified and correctly excluded tissue artifacts in 27% of images, reducing window area by a mean of 9.8 ± 8.2% relative to convex hull methods. Convex hull window misspecification inflated K function AUC by up to 649.6% and produced 100% false rejection of complete spatial randomness under simulation, errors fully corrected by PatternExtract. Cross-cohort validation on an independent 20-patient cohort confirmed pipeline consistency across imaging platforms and staining protocols. Biological application to Ki67^+^ cell distributions in DLBCL demonstrated the pipeline’s utility in detecting genuine short-range clustering beyond spatial inhomogeneity. PatternExtract is available at https://github.com/shrutisridhar99/PatternExtract with a Streamlit-based graphical user interface (GUI) for accessibility across programming backgrounds.

## Graphical Abstract

**Figure:**
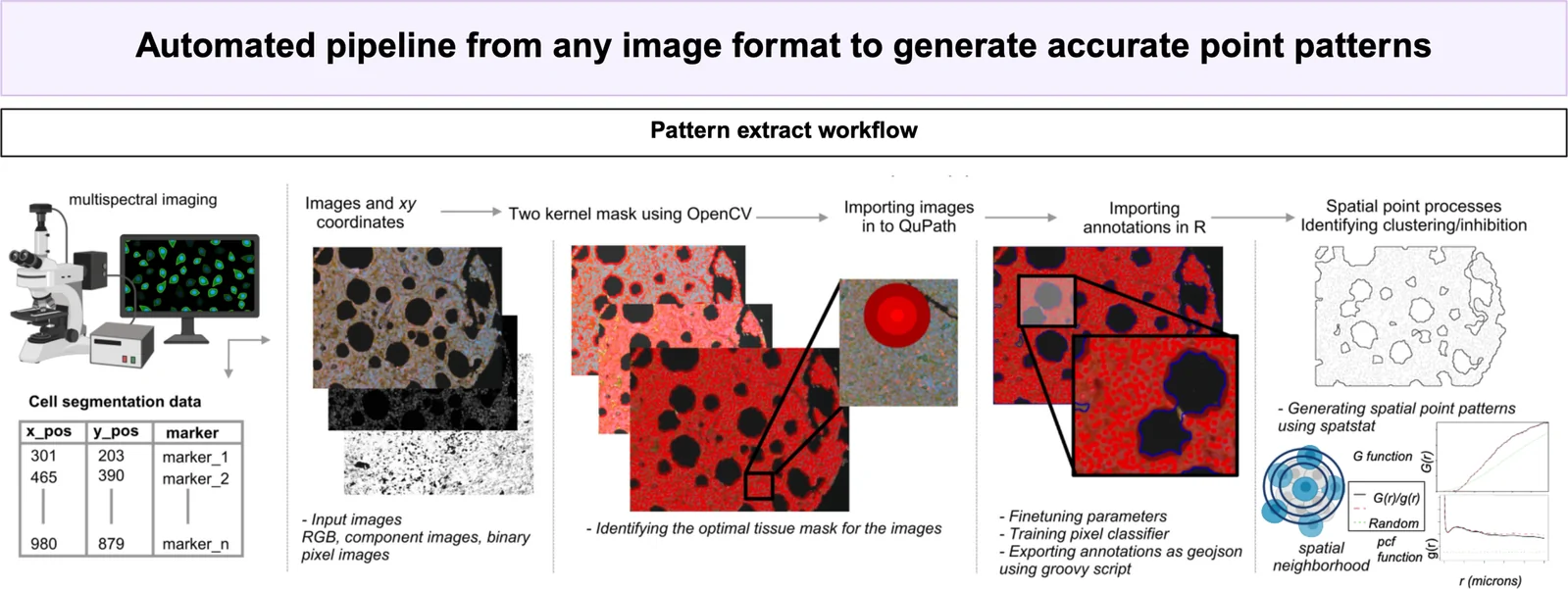

## Introduction

Spatial biology is increasingly integral to cancer research. With the advent of methods to provide molecular information at single cell resolution and preserving spatial detail in sections of cancer tissue, it is now possible to evaluate inter-relationships between malignant cells and the tumor microenvironment (TME). Most published spatial analyses in cancer rely on nearest neighbor distance measures, which provide a simplified, global summary of cell proximities. However, such aggregated measures may obscure spatial heterogeneity in spatial distribution of cells within the region of interest (ROI) and limit biological insight.

A more robust approach is through spatial point pattern analysis, a mathematical framework used to study the relationships between data points in 2-dimensional space. This method is well-established in fields such as ecology [1–3], where it is employed to examine interactions between plant species, and in geographic information systems (GISs) to quantify spatial relationships between geographic features. Representing data as 2- or 3-dimensional spatial point patterns enables the application of rigorous mathematical functions in various spatiotemporal studies. These methods can quantitatively assess deviations from complete spatial randomness (CSR) [2] modeled mathematically as a Poisson distribution and help to infer whether spatial features are randomly distributed, ordered, or clustered.

Drawing from the utility of spatial point pattern analysis in ecology, these approaches are potentially valuable to understand the ecosystem of cancer using multiplexed immunohistochemistry (mIHC) or multiplexed immunofluorescence (mIF) data, which provide spatial coordinates for biomarkers at single-cell resolution. Current approaches typically rely on tools such as QuPath [4], SpaceC [5], or Cellpose-SAM [6] to extract cell coordinates and phenotype cells. A comparison of key capabilities across common tools is provided in Table 1. The outputs are subsequently processed using the geospatial R package spatstat [1]. This package offers a comprehensive set of tools to fit spatial point process models, enabling the quantitative analysis of spatial organization within the TME. For instance, multi-scale modeling of cellular inhibition and regularity within the TME, such as homotypic cell inhibition, has been implemented using spatstat [7,8]. Complementary tools for spatial analysis of multiplexed imaging data have also been developed, including ALOA [9], which provides spatial profiling analysis from InFORM-derived coordinate data, SNAQ [10], which enables neighborhood analysis from QuPath-derived coordinates, and Histolytics [11], which offers whole slide image (WSI)-scale histopathological analysis with spatial querying capabilities. However, the accurate inference of spatial point patterns critically depends on the definition of the region within which the point pattern is analyzed, known as the spatial window.

**Table 1.** Comparison of existing cell segmentation and annotation tools, highlighting limitations addressed by PatternExtract

Tool	Type	Handles holes/folds	RGB compatible	Outputs GeoJSON/CSV	Open-source
Cellpose	Cell segmentation	✖	✖	✖	✔
QuPath	Annotation	Partial	✖	✔	✔
Visiopharm	Cell segmentation + Annotation	✖	✖	✖	✖
PatternExtract	Tissue + Point mask	✔	✔	✔ (through QuPATH)	✔

Existing boundary functions often approximate the ROI using convex hulls or rectangular windows, which may include artifacts such as blood vessels, necrotic zones, or tissue damage, posing a challenge for accurate spatial analyses. Alpha-hulls and concave hulls can capture nonconvex shapes but fragment into disconnected components at the resolution required to detect tissue voids. Kernel density estimation generates smooth continuous surfaces but cannot produce discrete binary tissue boundaries. Further, access to TIFF images is often difficult and tools lack the capabilities to work with lower-resolution image formats supplemented with pathologist annotations.

The pipeline developed in this study addresses these limitations by applying a 2-kernel mask method to the spatial coordinates of cells in each image, accurately defining spatial windows that exclude noncellular regions such as necrosis, tissue loss, or artifacts. This multi-platform workflow is compatible with diverse image modalities (including whole-slide, RGB, and fluorescence images) and can incorporate cell-type annotations from any segmentation tool to generate spatial point patterns using the 2-kernel mask and spatstat packages. In addition, the pipeline leverages QuPath, an open-source digital pathology platform, for visualization and user validation of images and annotations while automating pixel classification to adaptively identify and exclude artifacts. To demonstrate its applicability, we developed and optimized this pipeline on a large cohort of 568 images from 274 patients of diffuse large B cell lymphoma (DLBCL). The pipeline consistently generated accurate spatial point patterns, independent of marker type. To validate its generalizability and explore biological applications, we applied the pipeline to an independent set of 20 images (*n* = 20 patients) from another cohort, confirming its robustness across diverse datasets and its utility for spatial analyses and TME studies in DLBCL.

## Methods

### Samples and dataset

Samples from patients diagnosed with DLBCL (*n* = 274) in tissue microarray (TMA) format were obtained from British Columbia Cancer Agency (BCA) [12]. Additional DLBCL TMAs were obtained from the National University Hospital (NUH) [approved by the Singapore NHG Domain Specific Review Board B study protocol (2015/00176)] (*n* = 20) and are available as test data on the codebase. Samples were obtained through Institutional Review Board (IRB)-approved ethics protocols, with written informed consent from the patients, or with IRB-approved waivers of consent where applicable in accordance with the ethical guidelines of the Declaration of Helsinki. Material transfer agreements from all providing institutions were incorporated into the framework of an NUS IRB-approved translational study (H-19-055E).

### Immunofluorescence staining

Quantitative multiplex fluorescence immunohistochemistry was performed using sequential opal tyramide signal amplification technology (OPAL-TSA) staining (Tables 2 and 3). Images were acquired using the Vectra 2 imager and analyzed using InFORM [13]. 4′,6-Diamidino-2-phenylindole (DAPI) nuclear staining and CD20 membrane staining were used to segment cells. The mean membrane intensity per cell was captured for CD20, and the mean nuclear intensity per cell was captured for Ki67. For each image, cells with a marker intensity above a given intensity threshold for that image were declared to be positive for that marker. A pathologist manually inspected each image to determine a reliable threshold for each marker that resulted in minimal false-positive and false-negative assignments. The cohorts were evaluated in a TMA format; depending on tissue availability and between 1 and 9 high-power 700 × 500 μm imaging fields were captured and evaluated per patient. Following this, the spatial pipeline was applied to derive spatial point patterns.

**Table 2.** Manual multiplexed fluorescent immunohistochemistry (mfIHC) staining protocol for the NUH cohort tissue microarray

Procedure	Reagent (product; dilution)	Duration (min)
1st primary antibody incubation	Anti-Bcl-6 (Leica, LN22, NCL-564; 1:30)	60
1st secondary antibody incubation	Anti-Mouse HRP (Agilent Cat# K4001, RRID: AB_2827819)	10
1st signal amplification and fluorophore deposition	TSA Opal 670 (PerkinElmer FP1487001KT; 1:100)	10
2nd epitope retrieval (antibody stripping)	pH 9 HIER (low power)	10
2nd epitope blocking	BSA	10
2nd primary antibody incubation	Anti-Bcl-2 (Agilent Cat# M0887, RRID: AB_2064429; 1:50)	60
2nd secondary antibody incubation	Anti-Mouse HRP	10
2nd signal amplification and fluorophore deposition	TSA Opal 520 (PerkinElmer FP1487001KT; 1:100)	10
3rd epitope retrieval (antibody stripping)	pH 9 HIER (low power)	10
3rd epitope blocking	BSA	10
3rd primary antibody incubation	Anti-c-Myc (Abcam Cat# ab32072, RRID: AB_731658; 1:50)	30
3rd secondary antibody incubation	Anti-Rabbit HRP (Agilent Cat# K4003, RRID: AB_2630375)	10
3rd signal amplification and fluorophore deposition	TSA Opal 570 (PerkinElmer FP1487001KT; 1:100)	10
4th epitope retrieval (antibody stripping)	pH 9 HIER (low power)	10
4th epitope blocking	BSA	10
4th primary antibody incubation	Anti-CD20 (Agilent Cat# M0755, RRID: AB_2282030; 1:2,000)	30
4th secondary antibody incubation	Anti-Mouse HRP	10
4th signal amplification and fluorophore deposition	TSA Opal 540 (PerkinElmer FP1487001KT; 1:100)	10
5th epitope retrieval (antibody stripping)	pH 9 HIER (high power)	5
5th epitope retrieval (continued)	pH 9 HIER (low power)	10
5th epitope blocking	BSA	10
5th primary antibody incubation	Anti-Ki67 (Agilent Cat# M7240, RRID:AB_2142367; 1:50)	45
5th secondary antibody incubation	Anti-Mouse HRP	10
5th signal amplification and fluorophore deposition	TSA Opal 620 (PerkinElmer FP1487001KT; 1:100)	10
Final antibody stripping	pH 9 HIER (low power)	10
Counterstaining	DAPI (PerkinElmer FP1490; 1:10 in antibody diluent Dako DKO.S302283)	5
Mounting	CC mount (Sigma C9368)	10

NUH, National University Hospital; TMA, tissue microarray; HRP, horseradish peroxidase; TSA, tyramide signal amplification; HIER, heat-induced epitope retrieval; BSA, bovine serum albumin; DAPI, 4′,6-diamidino-2-phenylindole

**Table 3.** Automated multiplexed fluorescent immunohistochemistry (mfIHC) staining protocol for the BCA cohort tissue microarray

Procedure	Reagent (product; dilution)	Duration (min)
Bake at 60 °C		20
Dewax	Leica Biosystems Bond Dewax Solution (AR9222)	BondMax default step
1st epitope retrieval (BondMax)	Leica Biosystems Bond Epitope Retrieval Solution 2 (AR9640)	20
Peroxide block	BOND Polymer Refine Detection Kit (DS9800)	10
1st primary antibody incubation	Anti-c-Myc (Abcam Cat# ab32072, RRID: AB_731658; 1:50)	20
1st post primary	BOND Polymer Refine Detection Kit (DS9800)	8
1st polymer	BOND Polymer Refine Detection Kit (DS9800)	8
1st signal amplification and fluorophore deposition	TSA Opal 520 (PerkinElmer FP1487001KT; 1:100)	10
2nd epitope retrieval (BondMax)	Leica Biosystems Bond Epitope Retrieval Solution 1 (AR9961)	15
2nd primary antibody incubation	Anti-CD20 (Agilent Cat# M0755, RRID: AB_2282030; 1:1,000)	20
2nd post primary	BOND Polymer Refine Detection Kit (DS9800)	8
2nd polymer	BOND Polymer Refine Detection Kit (DS9800)	8
2nd signal amplification and fluorophore deposition	TSA Opal 540 (PerkinElmer FP1494001KT; 1:100)	10
3rd epitope retrieval (BondMax)	Leica Biosystems Bond Epitope Retrieval Solution 1 (AR9961)	20
3rd primary antibody incubation	Anti-Bcl-6 (Leica, LN22, NCL-564; 1:50)	20
3rd post primary	BOND Polymer Refine Detection Kit (DS9800)	8
3rd polymer	BOND Polymer Refine Detection Kit (DS9800)	8
3rd signal amplification and fluorophore deposition	TSA Opal 620 (PerkinElmer FP1495001KT; 1:100)	10
4th epitope retrieval (BondMax)	Leica Biosystems Bond Epitope Retrieval Solution 2 (AR9640)	20
4th primary antibody incubation	Anti-Bcl-2 (Agilent Cat# M0887, RRID: AB_2064429; 1:100)	20
4th post primary	BOND Polymer Refine Detection Kit (DS9800)	8
4th polymer	BOND Polymer Refine Detection Kit (DS9800)	8
4th signal amplification and fluorophore deposition	TSA Opal 690 (PerkinElmer FP1497001KT; 1:100)	10
Counterstaining	DAPI (PerkinElmer FP1490; 1:10 in antibody diluent Dako DKO.S302283)	5
Mounting	ProLong Diamond Antifade Mountant, CC mount (Life Technologies)	

BCA, British Columbia Cancer Agency; TMA, tissue microarray; HRP, horseradish peroxidase; TSA, tyramide signal amplification; HIER, heat-induced epitope retrieval; BSA, bovine serum albumin; DAPI, 4′,6-diamidino-2-phenylindole

### RGB export for spatial analysis:

Following multispectral image acquisition and unmixing, RGB composite images were exported specifically for spatial analysis. It is important to note that during RGB export, inFORM converts the spectrally unmixed single-channel TIFFs into a 3-channel color image solely for visualization. As a result, the original fluorophore channel assignments are not preserved in the RGB file; instead, the software maps selected channels into red, green, and blue.

### Generating tissue segmentation masks

Spatial windows can be generated for any image that has an accurate segmentation mask. To build such a mask using the RGB images and spatial coordinates, we used the image processing package OpenCV [14]. The pipeline can be generalized to all images if the coordinates with marker information are provided as a csv file. Our method overlays points on the RGB images, factoring in how the coordinates scale with reference to the image.

For each cell centroid (x_i_, y_i_), 2 binary circular kernels are defined. D₀(x; x_i_) is a filled disk of effective radius ~7 px (thickness/2) centered at x_i_ with intensity 255, implemented via cv2.circle() with radius = 0 and thickness = 15. D₅(x; x_i_) is a binary annular kernel with inner radius 5 px and outer radius ~20 px (inner radius + thickness), intensity 200, implemented via cv2.circle() with radius = 5 and thickness = 15. The tissue map M(x) = 0.5·D₀(x; x_i_) + 0.5·D₅(x; x_i_) is generated by additive blending via cv2.addWeighted() with equal weights. In cell-dense regions, overlapping kernels from adjacent cells sum to produce higher intensity values. Both kernel parameters (radius and thickness) are tunable and can be adjusted for different tissue types or imaging resolutions using the provided utility function (utils/recommend_radius.py), which computes the cohort-specific nearest-neighbor distance distribution and recommends an optimal kernel radius for any new dataset.

### Pixel classifier in QuPath for generating annotation: Fine tuning parameters

The QuPath tool includes a built-in pixel classifier that can be trained on images to generate tissue segmentation masks. The classifier uses the create thresholder function, along with tunable parameters such as minimum object size, minimum hole size, a residual channel with a Gaussian prefilter, and the full-resolution setting (downsample = 1) to produce GeoJSON annotations. During training, the threshold categories “Unclassified” and “Region*” are used to annotate the entire image. These GeoJSON annotations contain a dictionary list of XY coordinates, which can then be imported to define spatial windows that accurately capture the tissue boundaries and accommodate heterogeneity across tissue sections. The pixel classifier applies Gaussian smoothing (σ = 5 px) followed by thresholding at −0.01 on the residual stain channel. Annotations are generated with minimum object size 1,350 px^2^ and minimum hole size 500 px^2^, followed by hole filling, and exported as GeoJSON polygon coordinates. After training, the classifier is first tested on a single image. Based on the optimized parameters, a Groovy script is generated to apply the classifier across the entire project and to automate the export of the GeoJSON annotations. The resulting annotated images are manually reviewed to verify that excluded regions are correctly identified and not misclassified. When necessary, the “Fill Holes” function can be applied to correct any erroneous exclusions in the annotations.

### Performance evaluation of spatial pipeline

The performance of the pipeline can be visually assessed by the accuracy of the QuPath annotations in the viewer. PatternExtract is a downstream module that operates on already segmented cells and tissue masks. Its primary function is to create standardized coordinate point patterns for spatial statistical analyses. In this context, the marks (cell coordinates) are the relevant analytical unit (previously generated upstream by any cell segmentation tool), and accurately placing them within valid tissue regions is the key metric. A quantitative measure of this performance was tabulated by calculating the percentage of points lying outside the spatial boundary imported from the QuPath annotations. This reflects the quality of the tissue mask and thereby the accuracy of annotations. In some cases, the annotations imported into the R script for replacing the default spatial point pattern window can have a scaling issue such that a shift in the mask is seen for a few images. This was addressed by replacing the scaling factor applied to adjust the default window xy min–max limits relative to the *xy* limits of the new spatial window. This can be seen in Fig. S2A, and the improvement with the new scaling can be seen in Fig. S2B (right). The number of images with points excluded also decreased with the new scaling (Table 4).

**Table 4.** Distribution of point deviations exceeding 1% and 5% thresholds before and after scaling correction in the BCA cohort

Dataset	Number of images	Number of points > 1% < 5%	Number of points >5%
BCA (before scaling)	568	68	1
BCA (after scaling)	568	0	0

### Spatial pipeline standalone package

The workflow used for generating spatial point patterns consists of the following parts: the python script using the OpenCV package to manipulate the kernel size and intensity of the spatial coordinates overlaid on the images; a QuPath groovy script to automate the import and export of the images into the project folder for QuPath and the parameter thresholds for the pixel classifier; an R script to build the spatial point patterns with the accurate GeoJSON annotations exported from QuPath. For the scientific community consisting of cancer biologists, pathologists, and clinicians with limited domain knowledge in programming, the individual steps of the abovementioned spatial analysis scripts have been consolidated into a standalone package that can be executed in a facile manner. The package supports the visualization of the images with the annotations through a pop-up QuPath window. The user can tweak and refine the annotations built by the classifier in QuPath at this intermediate step. The pipeline can be run on any image with a corresponding csv file containing the spatial coordinates. We built a Streamlit-powered interactive web application designed to aid in easy access to the pipeline. The platform integrates image processing, QuPath automation, and R-based spatial statistics into a unified, user-friendly web interface. Upon launch, the application automatically detects the operating system (Windows, macOS, or Linux) and attempts to identify the project’s base directory containing the required structure (data/CSV, data/Images_RGB, and scripts). The user is allowed to override auto-detected paths via the sidebar. If enabled, the tool launches QuPath in noninteractive (console) mode to perform project creation, pixel classification, and object segmentation. The pipeline supports both Windows [QuPath-0.6.0 (console).exe] and macOS (QuPath-0.6.0-arm64). Processed statistics are displayed using custom metric cards in the Results section (Fig. S2C).

### Code availability and reproducibility

The complete PatternExtract codebase is available at https://github.com/shrutisridhar99/PatternExtract. A requirements.txt file with pinned dependency versions is provided for reproducible installation. Computational performance was benchmarked on 20 NUH images on a standard consumer laptop (Apple M-series, CPU only, no GPU). Python mask generation required 1.63 s per image (peak memory 57.2 MB), QuPath annotation required 2.4 s per image, and R spatial statistics required 0.7 s per image, giving a total of 4.8 s per image and an estimated runtime of approximately 45 min for 568 images. All 3 steps are parallelizable at the image level.

## Results

### Two-kernel tissue segmentation masks for TMA images

**Fig. 1.**
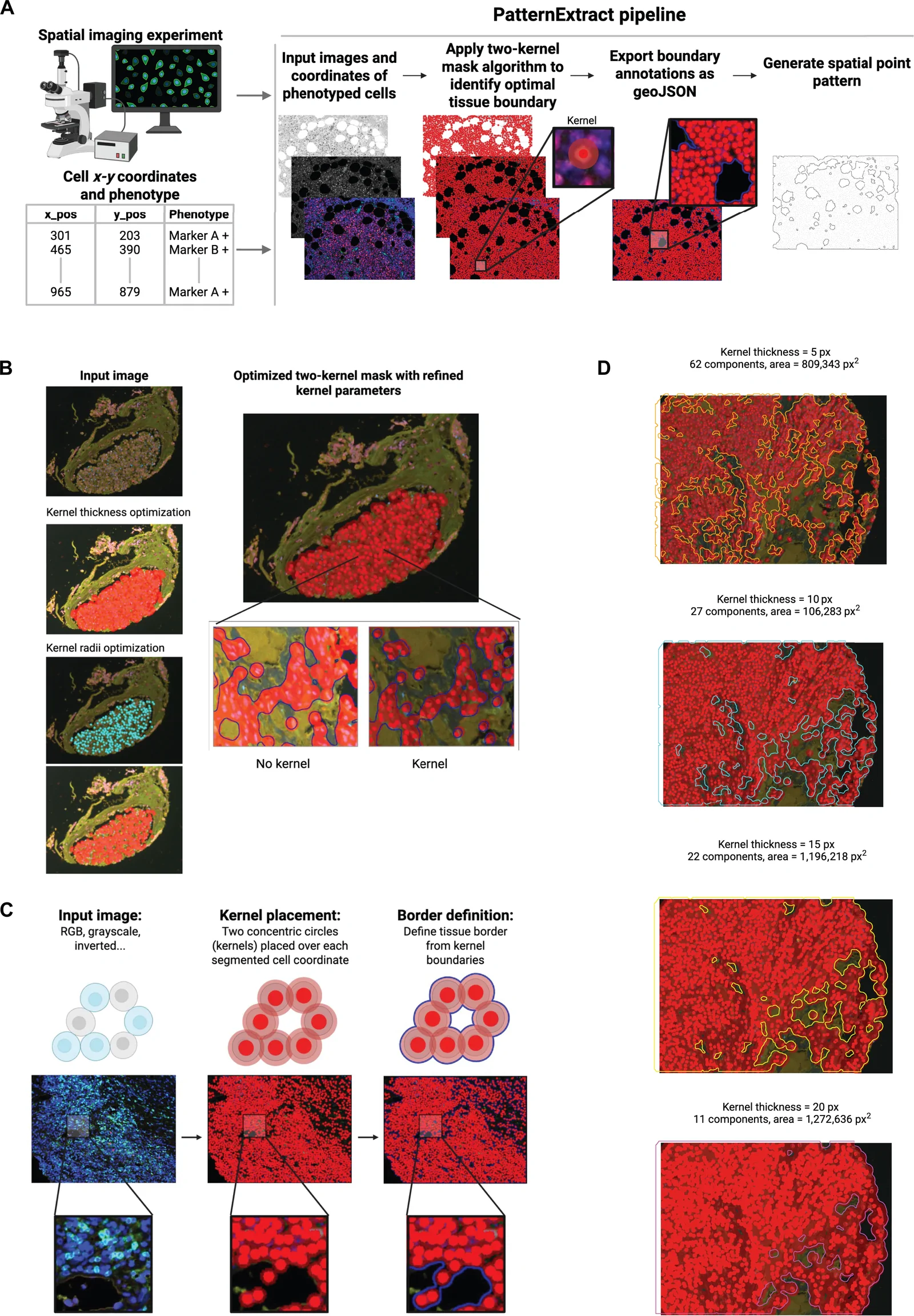
Overview of the PatternExtract pipeline for generating spatial point patterns from multiplex imaging data. (A) Representative RGB input image from multiplex immunofluorescence-stained DLBCL tissue and PatternExtract pipeline overview. (B) Two-kernel overlay at different radii showing iterative parameter optimization. (C) Final optimized 2-kernel mask with zoomed inset confirming accurate delineation of tissue boundaries at cellular resolution. (D) Kernel sensitivity analysis: number of connected components (left) and window area (right) as a function of kernel thickness (5, 10, 15, 20 px).

We generated tissue segmentation masks with a 2-kernel approach by overlaying the spatial coordinates on the 568 image cohort from BCA. This cohort consisted of RGB images of multi-fluorescence immunohistochemistry without an available inFORM tissue segmentation mask. Only the red, green, blue channels are available for the RGB images when viewed in QuPath as brightfield fluorescence. Overlaying the coordinates was a starting point, and after several iterations of point size and radii, we arrived at the 2-kernel approach to define each cell as 2 concentric circles (Fig. 1B). This method operates by first mapping cellular XY coordinates onto the tissue sections and then systematically expanding each coordinate point to construct the tissue mask. The expansion architecture comprises a central kernel region encompassed by 2 concentric circular kernels of varying radii, with differential shading applied to preserve visualization of the underlying XY coordinate overlay within the mask boundaries. The resulting masks were subsequently converted to GeoJSON format for precise tissue boundary geometries, enabling importation into R for downstream spatial statistical analysis. The larger circle with lower red channel intensity circumscribes the smaller circle with higher intensity (Fig. 1C). This approach defined the tissue contours and the single cell positions without dependence on proprietary software and can take the input from any cell segmentation tool.

The effective kernel thickness of the 2-kernel approach (*r* = 15 px) was independently validated by sensitivity analysis across radii of 5, 10, 15, and 20 px on the same representative image (Fig. 1D). The number of connected components decreased rapidly from 62 (*r* = 10 px) to 22 (*r* = 15 px) to 11 (*r* = 20 px), with the rate of decrease slowing markedly beyond *r* = 15 px, identifying *r* = 15 px as the elbow point of the connectivity curve (Fig. S1A). Window area increased steadily from *r* = 10 px to *r* = 15 px and then rose sharply at *r* = 20 px, indicating that larger radii begin bridging genuine tissue gaps (Fig. S1B). Together, these criteria independently identify *r* = 15 px as the minimum radius thickness that achieves connected tissue representation without over-expansion, corresponding to approximately one intercell spacing (median nearest-neighbor distance 14.4 ± 1.7 px across 568 BCA images).

**Fig. 2.**
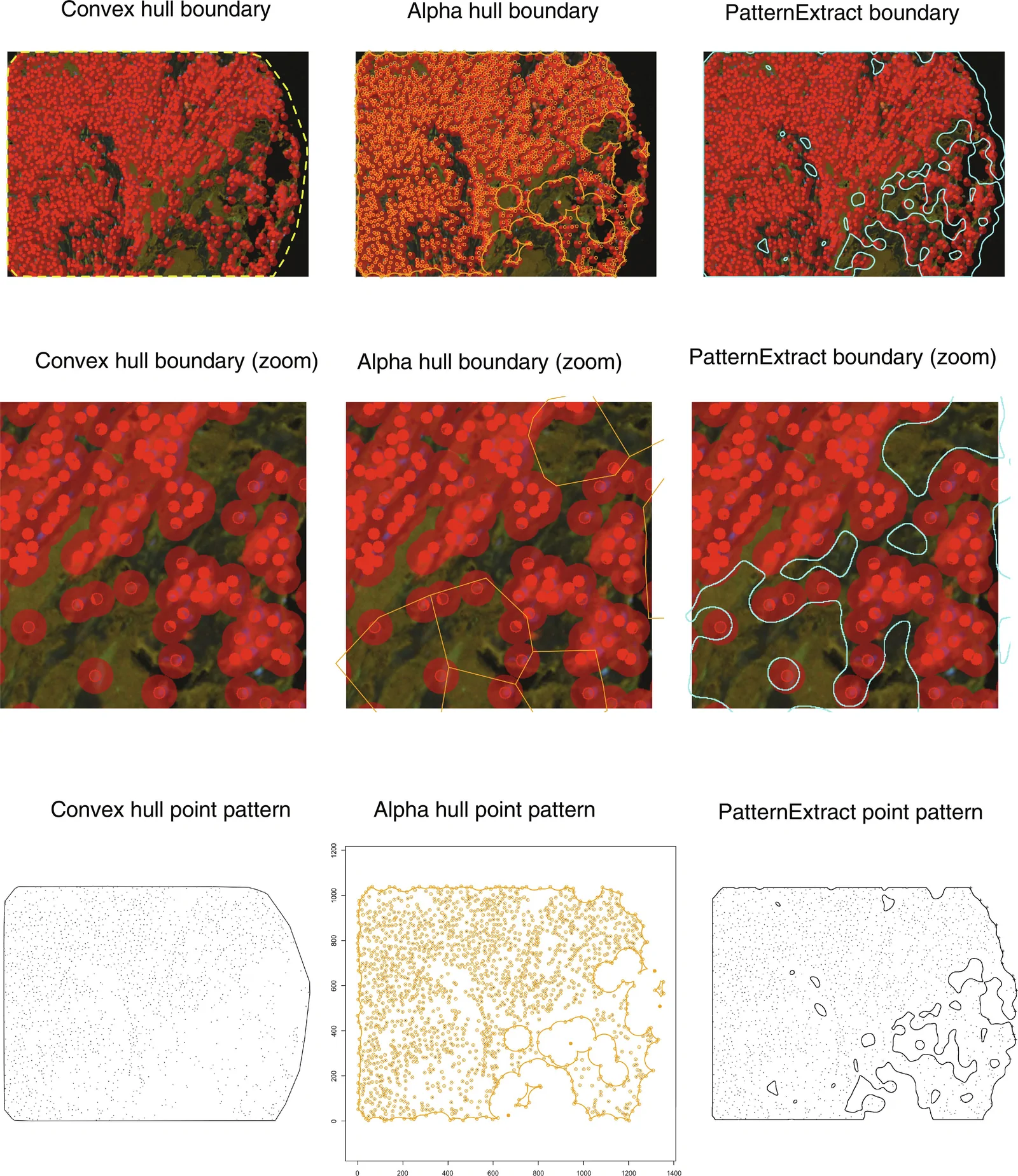
Comparison of boundary estimation methods on a representative DLBCL TMA image (A1_02_04). (A) Full image view showing convex hull boundary (yellow dashed), alpha-hull boundary (orange), and PatternExtract boundary (cyan). The convex hull includes all void regions; the alpha-hull fragments into disconnected per-cell halos; PatternExtract produces a single connected boundary correctly excluding tissue voids. (B) Zoomed panels showing the same boundary at cellular resolution, confirming accurate tracing of tissue edges. (C) Point patterns under each method are shown; compared to other methods, the PatternExtract window yields a biologically interpretable spatial region suitable for downstream spatial statistics.

We compared PatternExtract against 2 alternative boundary estimation methods on a representative image containing tissue voids (A1_02_04, 10.3% area reduction; Fig. 2). The convex hull produced a single outer boundary that included all void regions (area = 1,335,430 px^2^; Fig. 2A and C, left). The alpha-hull (alpha = 50) fragmented into per-cell halos at the resolution required to detect voids, producing thousands of disconnected regions unsuitable for spatial statistics (Fig. 2A and C, middle). PatternExtract produced a single connected boundary correctly excluding all major void regions (area = 1,198,193 px^2^; Fig. 2A and C, right), representing the only method yielding a biologically interpretable spatial window suitable for downstream statistical analysis. Zoomed panels (Fig. 2B) confirm that the PatternExtract boundary correctly traces individual tissue edges at cellular resolution.

### Tissue artifacts: Tissue damage and innervating blood vessels

DLBCL TMA sections exhibit inherent heterogeneity including clumps of cells interspersed with acellular regions, tissue loss with structural holes, and innervating blood vessels producing circular acellular zones. The reddish orange background signal of RGB images in acellular regions presents a challenge for the pixel classifier, as overly large minimum object size parameters exclude genuine cell clusters, while overly permissive settings retain background artifacts. We addressed this by converting RGB images to grayscale and optimizing minimum object and hole size parameters (Fig. S1C), producing accurate tissue boundaries across heterogeneous tissue morphologies.

**Fig. 3.**
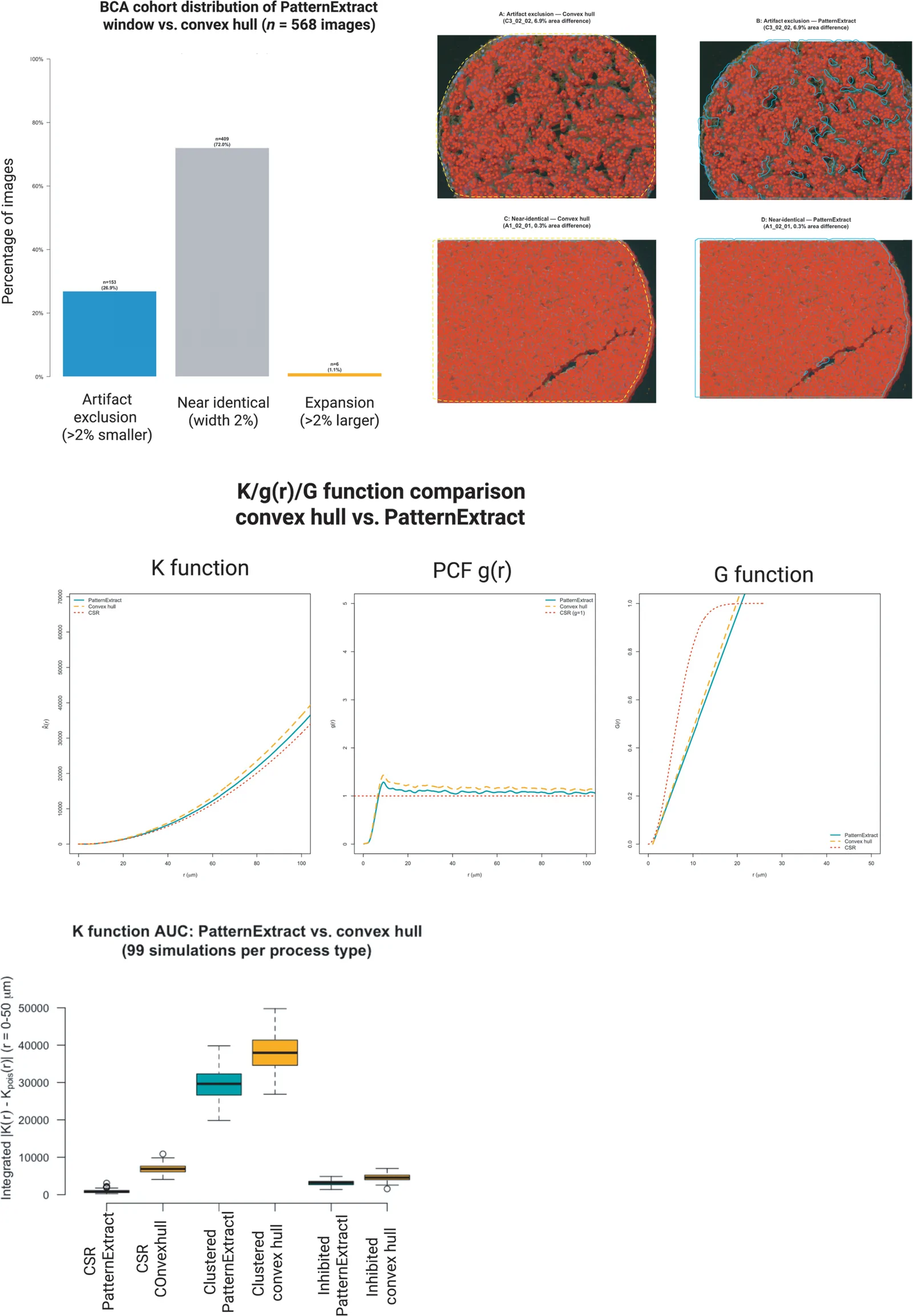
Evaluation of window quality (A) Quantitative evaluation of PatternExtract window quality across the BCA cohort (*n* = 568 images). (B) Representative example of artifact exclusion (C3_02_02, 6.9% area reduction): Convex hull (left) includes a fibrotic void region; PatternExtract (right) correctly excludes it. Bottom row: Representative near-identical example (A1_02_01, 0.3% difference): Both methods produce equivalent boundaries on a clean core, confirming that PatternExtract does not over-exclude tissue in artifact-free sections. (C) Deviation of convex hull from PatternExtract for K function (left), g(r) (middle), and G function (right) for representative image A1_02_01. (D) K function AUC comparison across 99 simulations each of CSR, Thomas cluster, and hard-core inhibited processes under PatternExtract versus convex hull windows. The convex hull inflates K function AUC by 649.6%, 28.0%, and 46.8% for CSR, clustered, and inhibited processes, respectively, demonstrating that window misspecification introduces consistent upward bias regardless of the true underlying spatial process.

Across the BCA cohort (*n* = 568), PatternExtract window area differed from the convex hull in 3 ways (Fig. 3A). In 153 images (27%), PatternExtract identified and excluded genuine tissue artifacts, producing a window that was, on average, 9.8 ± 8.2% smaller than the convex hull (max 38.6%), as exemplified by the representative image in Fig. 3B (top), where the convex hull incorrectly includes a large fibrotic void region, while PatternExtract correctly excludes it. In 409 images (72%), the 2 windows were near-identical (within 2%), confirming that PatternExtract does not over-exclude tissue in artifact-free sections, as shown in image A1_02_01 where both methods produce equivalent boundaries (Fig. 3B, bottom). In 6 images (1%), the PatternExtract window was marginally larger than the convex hull (mean 3.4%, max 4.4%).

The downstream impact of window misspecification was quantified directly. The convex hull underestimated cell density (λ) by a mean of 3.1 ± 8.3% across 568 images (max 63.0%), because nontissue area inflates the window denominator, which is a bias that propagates to all downstream spatial statistics. To quantify the impact on the K function, we computed the integrated deviation from the CSR expectation, a measure of how much apparent spatial structure is introduced by window choice alone, independent of any true biological signal. On image A1_02_04 (10.3% area reduction), this deviation AUC was 180,780 μm^2^ under the convex hull versus 83,789 μm^2^ under PatternExtract, a 115.8% inflation demonstrating that the convex hull introduced substantial spurious clustering signal even though the underlying cell distribution was identical under both windows. The convex hull also produced CSR envelopes 16.1% narrower at *r* = 50 μm (Fig. 3C, left), increasing the probability of falsely rejecting CSR. The PCF g(r) was systematically elevated above CSR under the convex hull at all distances (Fig. 3C, middle), while the G function was identical under both windows, consistent with the analytical result that nearest-neighbor distances are unaffected by window shape (Fig. 3C, right).

Simulation experiments confirmed these findings at scale (Fig. 3D). Under 99 CSR simulations, PatternExtract correctly retained CSR classification in 96.9% of cases (type I error 3.1%, consistent with the nominal 5% level), while the convex hull falsely rejected CSR in all 99 simulations (type I error 100%). Extended simulations across Thomas cluster and hard-core inhibited processes showed consistent K function AUC inflation of 649.6%, 28.0%, and 46.8%, respectively, under the convex hull, demonstrating that window misspecification introduces upward bias regardless of the true underlying spatial process.

Cross-cohort consistency was confirmed in the independent NUH cohort (*n* = 20). The proportion of images with artifact exclusion was 27% in BCA and 25% in NUH. The convex hull underestimated λ by 3.1 ± 8.3% in BCA and 4.9 ± 14.7% in NUH. Mean area reduction among affected images was 9.8 ± 8.2% in BCA and 16.6 ± 13.4% in NUH, strikingly consistent despite different imaging protocols, staining platforms, and segmentation software.

### Biological application of point patterns

**Fig. 4.**
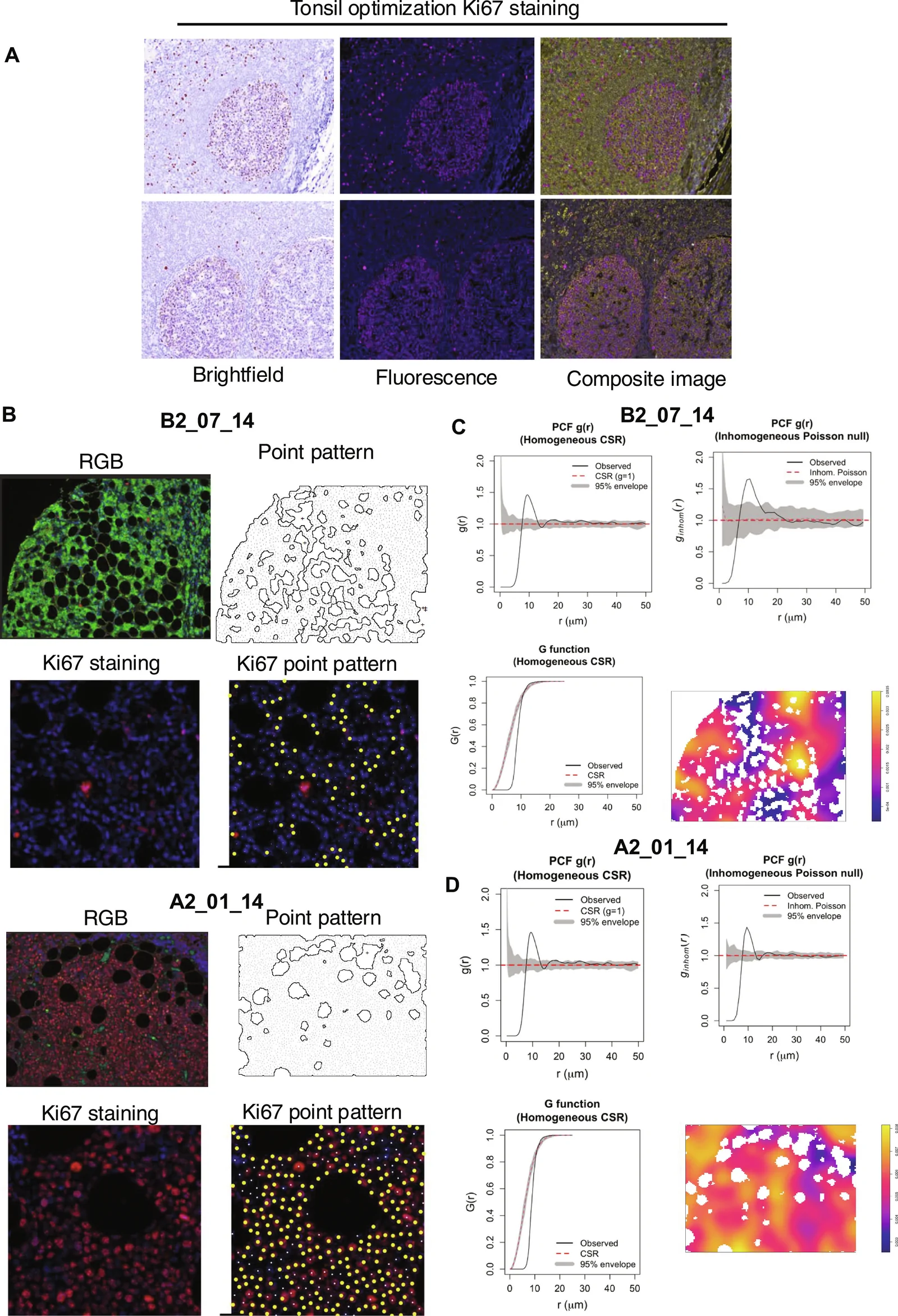
Biological application of spatial point pattern analysis to CD20^+^Ki67^+^ cells in DLBCL. (A) Optimization of Ki67 staining on tonsil tissue. (B) Two representative DLBCL regions (B2_07_14, top; A2_01_14, bottom) are shown with corresponding RGB multichannel images, nuclear/cell boundary point patterns, Ki67 fluorescence staining, and detected Ki67-positive cell point patterns (yellow/red dots). (C) Spatial statistics for sample B2_07_14. PCF [g(r)] under homogeneous CSR (left) and inhomogeneous Poisson null models (right). The G function (lower left) and the kernel density heatmap (lower right) visualizes local intensity of Ki67-positive cells. (D) Spatial statistics for sample A2_01_14. PCF [g(r)] under homogeneous CSR (left) and inhomogeneous Poisson null models (right). The G function (lower left) and the kernel density heatmap (lower right) visualizes local intensity of Ki67-positive cells.

We investigated the spatial distribution of Ki67-positive cells in DLBCL (*n* = 20) to illustrate the downstream utility of spatial point pattern analysis (Fig. 4A). Ki67, a well-established marker of proliferation, has been previously studied as a prognostic factor in DLBCL [15,16]. To assess its spatial organization, we employed point pattern analysis with a focus on deviations from CSR, using the pair correlation function (PCF) [17]. Across the NUH cohort, the mean Ki67^+^ fraction was 64.9% of CD20^+^ cells per image (77,358 total cells analyzed).

Two example DLBCL images and their respective identification of Ki67-positive cells are shown in Fig. 4B. Monte Carlo simulation envelopes (99 simulations) were generated for all spatial statistics, with translation correction applied for the PCF and border correction for the G function. The observed PCF exceeded the upper simulation envelope at short distances in both images [B2_7_14: g(10 μm) = 1.47 versus envelope maximum = 1.05; A2_1_14: g(10 μm) = 1.43 versus envelope maximum = 1.08], confirming statistically significant short-range clustering of CD20^+^Ki67^+^ cells (Fig. 4C, top left, and D, top left). The G function fell below the lower envelope at short distances indicating short-range inhibition consistent with physical cell exclusion volumes (Fig. 4C, bottom left, and D, bottom left). These 2 findings are not contradictory, where cells form local clusters while maintaining a minimum intercellular spacing, reflecting multi-scale spatial structure.

To account for tissue heterogeneity, we estimated the spatial intensity surface of CD20^+^Ki67^+^ cells using kernel smoothing (σ = bw.diggle), revealing clear spatial heterogeneity in cell density across the tissue with high-density regions interspersed with acellular follicular voids correctly excluded by the PatternExtract window (Fig. 4C, bottom right, and D, bottom right). We then tested against an inhomogeneous Poisson null model simulating from this estimated intensity surface. In both images, short-range clustering in the PCF persisted above the inhomogeneous envelope at *r* = 8 to 10 μm [B2_7_14: g_inhom(10 μm) = 1.66 versus envelope maximum = 1.29; A2_1_14: g_inhom(10 μm) = 1.36 versus envelope maximum = 1.07; Fig. 4C, top right, and D, top right], confirming genuine biological short-range clustering that cannot be explained by spatial inhomogeneity alone. Consistent spatial patterns were observed across NUH images, confirming the reproducibility of the spatial analysis pipeline.

## Discussion

We introduce PatternExtract, a robust, open-source pipeline to generate biologically accurate spatial point patterns directly from RGB images and coordinate data. A key strength of PatternExtract lies in its ability to automate tissue mask generation using a 2-kernel approach that captures tissue geometry and density without relying on fluorescence composite channels or vendor-specific image formats. This feature is particularly advantageous in retrospective or collaborative studies where only RGB images and cell coordinate files are available. Moreover, by exporting high-fidelity GeoJSON annotations from QuPath, the pipeline mitigates the inclusion of nontissue artifacts and voids. The examples highlighted demonstrate the adaptability of the PatternExtract pipeline in processing varied tissue morphologies, which ultimately help in providing accurate spatial summary functions such as the PCF and G-function. PatternExtract was developed specifically on hematological tissue characterized by high cellularity and clearly defined vascular and necrotic voids. Several limitations apply when extending to other tissue types. In very sparse regions, pericellular kernels may not overlap sufficiently to form a connected mask, in this case, the reccomend_radius function would be useful to find the right kernel size. Under-segmentation may underrepresent tissue extent in regions of missed cells. If spatial analysis is restricted to a rare marker-positive subpopulation, the mask should be generated from all segmented cells rather than the subpopulation alone. Extension to whole-slide images requires image tiling, and mask stitching is identified as a priority for future development. Future iterations could extend support for batch inference of spatial interactions across multiple marker pairs, incorporate deep learning-based segmentation to further improve robustness, and integrate with spatial transcriptomics deconvolution frameworks such as SegDecon [18] for multimodal tissue analysis.

## Data Availability

The entire codebase and test data for the pipeline can be found at: https://github.com/shrutisridhar99/PatternExtract.
