# Supplementary material for: PatternExtract: A Facile, Scalable Pipeline for Point Pattern Generation from Spatial Imaging Data
Source: Comput Struct Biotechnol J. 2026 Sep 9;1(1):0141. doi: 10.34133/csbj.0141 (PMC13554418; doi:10.34133/csbj.0141)

Supp Fig 1

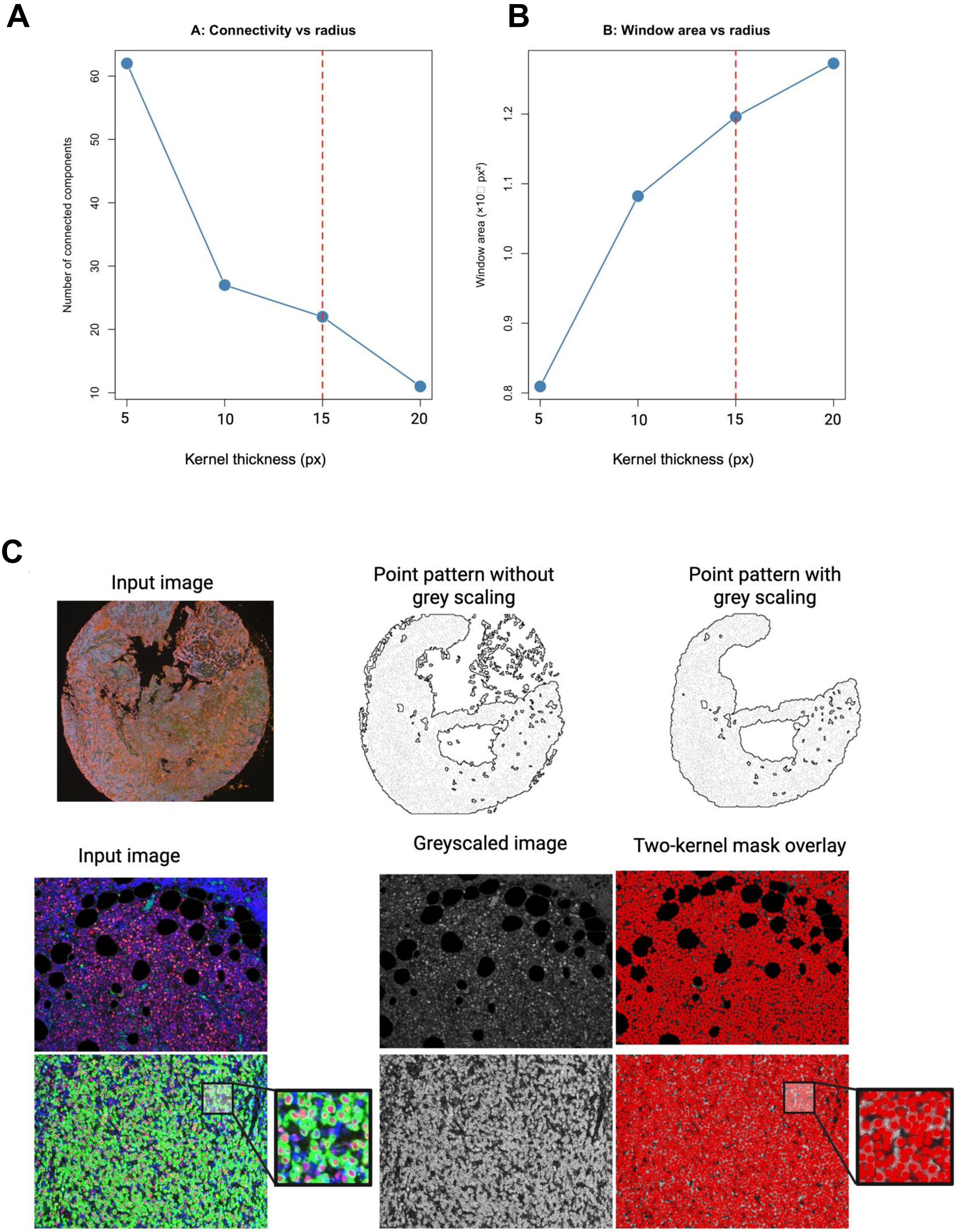

A
A: Connectivity vs radius
Number of connected components
10
20
30
40
50
60
5
10
15
20
Kernel thickness (px)
B
B: Window area vs radius
Window area (×10□ px²)
0.8
0.9
1.0
1.1
1.2
5
10
15
20
Kernel thickness (px)
C
Input image
Point pattern without grey scaling
Point pattern with grey scaling
Input image
Greyscaled image
Two-kernel mask overlay

Supp Fig 2

A

Shifting of boundary observed

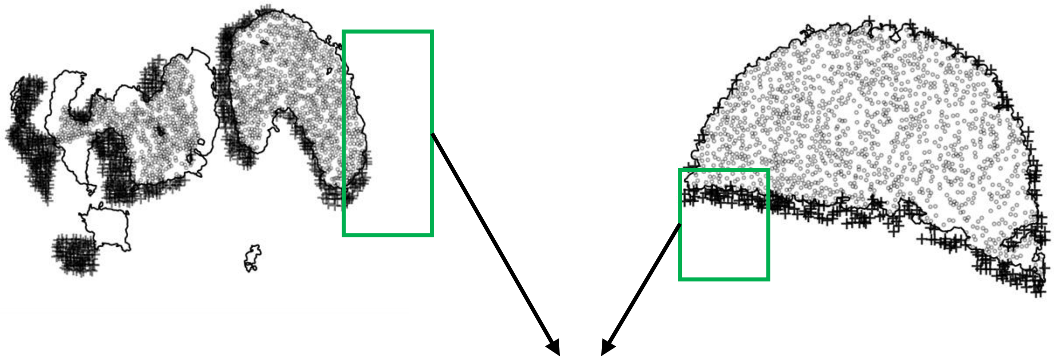

Often due to apparent shifting of the boundary, points can be excluded from the spatial point pattern object (denoted by ‘+’)

B

Fixed by changing scaling

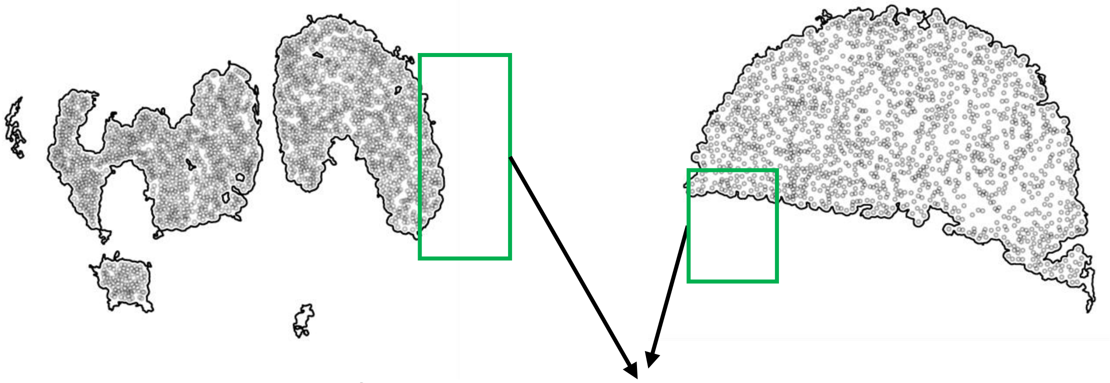

After scaling correction no points excluded.

C

PatternExtract GUI

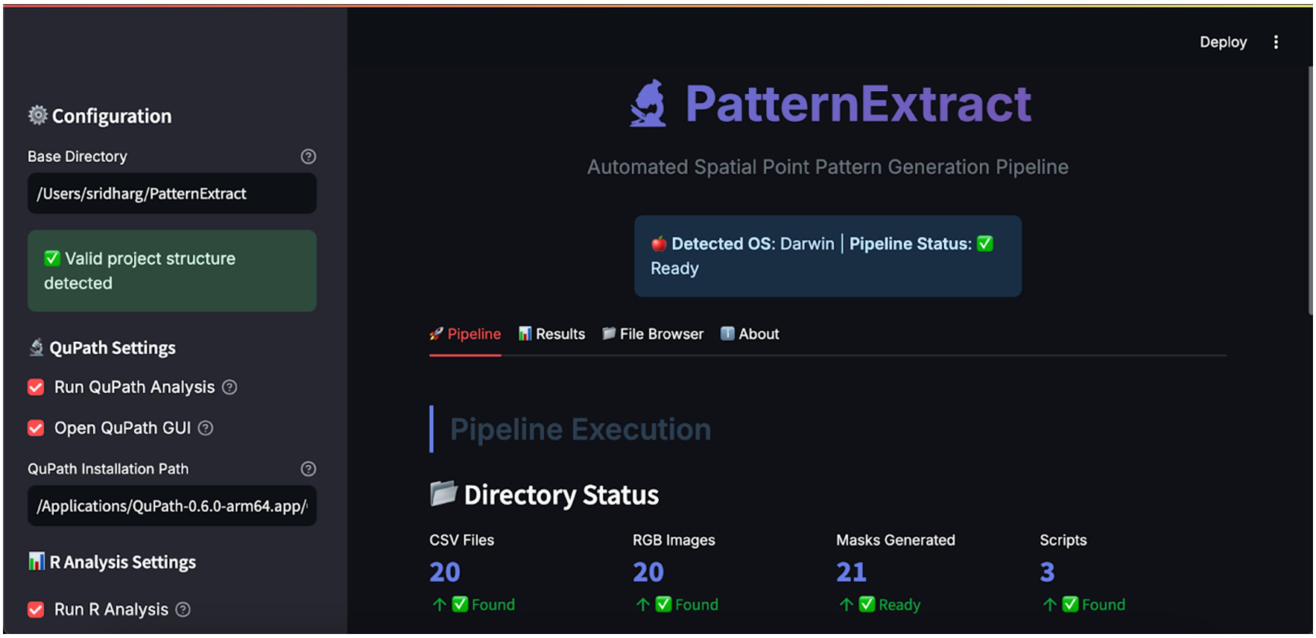

Supplement: Supplementary file 1 [file csbj.0141.f1.zip › Supplementary Figures.pdf]
