# Supplementary material for: PatternExtract: A Facile, Scalable Pipeline for Point Pattern Generation from Spatial Imaging Data
Source: Comput Struct Biotechnol J. 2026 Sep 9;1(1):0141. doi: 10.34133/csbj.0141 (PMC13554418; doi:10.34133/csbj.0141)
Supplement: Supplementary file 1 [file csbj.0141.f1.zip › Supplementary Labels.docx]

Supplementary Figure 1. Kernel Parameter Optimization and Tissue Segmentation Pipeline for Spatial Point Pattern Analysis

(A)Effect of kernel thickness on tissue connectivity. The number of connected components in the segmented tissue mask is plotted as a function of kernel thickness (5–20 px). Increasing kernel radius progressively reduces fragmentation, consolidating tissue regions into fewer connected components. The red dashed line at 15 px indicates the selected optimal kernel thickness, which balances adequate connectivity with preservation of tissue boundaries. (B) Effect of kernel thickness on analysis window area. Total window area (×10^4^ px^2^) is shown across the same range of kernel thicknesses. (C) Tissue segmentation and greyscale preprocessing workflow. (Top row) A whole-tissue section input image is shown alongside the resulting cell point patterns generated without (middle) and with (right) greyscale normalization prior to segmentation. Greyscaling substantially reduces spurious point detection in artifactual or non-cellular regions, producing a cleaner tissue outline. (Bottom rows) Representative high-magnification tissue images (left column) are shown with their corresponding greyscaled versions (middle column) and the two-kernel morphological mask overlay (right column, red), which delineates valid tissue area for point pattern analysis. Inset panels highlight the precision of the mask at single-cell resolution, demonstrating accurate exclusion of tissue voids and non-cellular regions (e.g., vessel lumina, artifacts).

Supplementary Figure 2. Boundary Shift Artifact Correction and PatternExtract GUI Overview

(A) Boundary shifting artifact in spatial point pattern construction. The excluded points are denoted by '+' symbols in the point pattern object. This artifact arises from coordinate scaling mismatches between the cell detection output and the tissue mask, leading to systematic exclusion of valid boundary-proximal cells from downstream spatial analyses. (B) Correction of boundary shift by scaling adjustment. (C) PatternExtract graphical user interface (GUI). Screenshot of the PatternExtract application.
